# Gastric Perforation With Peritonitis Secondary to a Trichobezoar: A Literature Review and Report of a Rare Presentation

**DOI:** 10.7759/cureus.24359

**Published:** 2022-04-21

**Authors:** Dharmendra K Pipal, Vijay Verma, murlidhar murlidhar, Garima Garima, Seema Yadav

**Affiliations:** 1 General, Colorectal and Minimal Access Surgery, All India Institute of Medical Sciences, Gorakhpur, Gorakhpur, IND; 2 General Surgery, Dr. Sampurnanand Medical College, Jodhpur, IND; 3 Pathology, Government Medical College, Pali, Pali, IND; 4 Anaesthesia, Rajmata Vijaya Raje Scindia Medical College, Bhilwara, IND

**Keywords:** trichobezoar, rapunzel syndrome, peritonitis, gastric perforation, trichophagia, trichotillomania

## Abstract

Trichobezoar, a rare disorder commonly seen in psychiatric patients having a habit of plucking and eating their own hair, is a ball of hair admixed with gastro-intestinal secretions that leads to the blocking of the passage of food particles. Presentation of the disease is variable, ranging from asymptomatic to severe complications including obstruction and perforation. We report a case of a 27-year-old female patient who presented with an acute abdomen and on laparotomy, gastric perforation secondary to large gastric trichobezoar was found. The patient was treated with en bloc removal of the trichobezoar.

## Introduction

Trichotillomania, a compulsive psychiatric disorder of hair pulling with a point prevalence of 0.5% to 6.5%, along with trichophagia, develops trichobezoar in 20-30% of cases [1-4]. With a mean age of 11 years and a sevenfold higher prevalence in children than in adults, the disease primarily affects females, with a peak in middle childhood and a minimum before the age of six years [5,6].

In approximately 80-90% of cases, foreign bodies escape from getting trapped in the stomach, but in trichobezoar, due to the slippery surface and sometimes with altered anatomy or physiology of the stomach, hairs get trapped in the mucosal folds of the stomach and form a hairball mixed with gastric secretions [7]. Rapunzel syndrome is a long trichobezoar when the bezoar extends or breaks off and migrates beyond the pylorus. Additional documented consequences include intussusception, obstructive jaundice/cholangitis, and pancreatitis [8].

As far as the complications are concerned, trichobezoar is frequently associated with gastric obstruction, but gastric perforation is extremely rare. When perforation occurs, this could be because there isn't enough blood flow to the stomach's mucous membrane, causing it to become ischaemic and bleed.

We report a case of gastric perforation secondary to gastric bezoar, an incidental and rare aetiology, presented in the emergency as perforation peritonitis with shock.

## Case presentation

**Figure 1:**
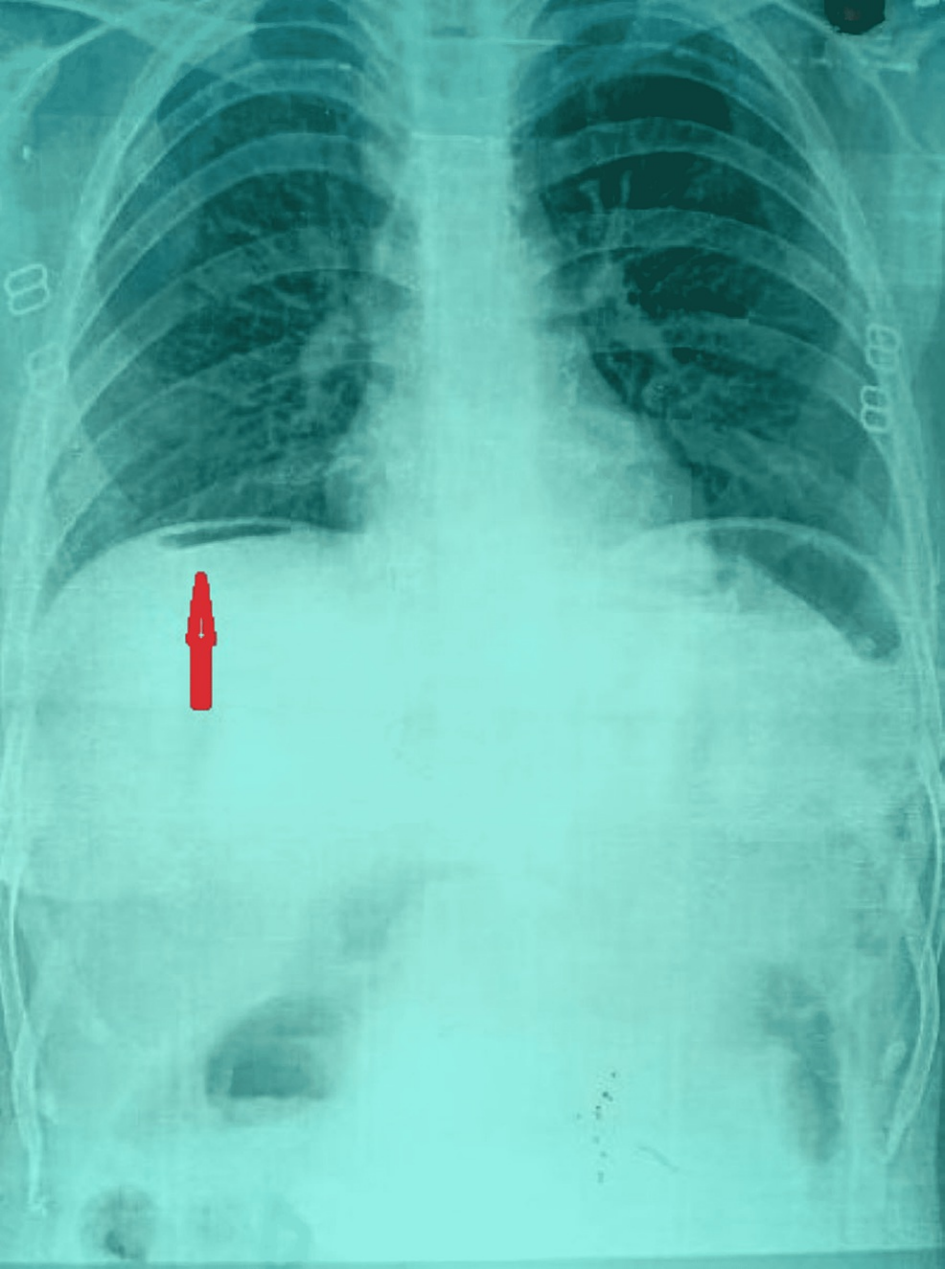
X-ray flat plate abdomen-standing, showing free air under the right dome of the diaphragm.

A 27-year-old female presented to the emergency department of our institution with complaints of abdominal pain, obstipation, and abdominal distension for five days. On examination, the patient was tachycardiac (PR = 108/min), febrile (101.2 °F), and had low blood pressure (90/56 mm Hg). On local examination, abdominal tenderness with generalised guarding and rigidity was present. After resuscitation, an X-ray abdomen was obtained, which revealed free gas under the right dome of the diaphragm (Figure 1). Mild to moderate amounts of free fluid with septations and coarse internal echoes were noted on ultrasonography of the abdomen. The patient's attendants also revealed a history of occasional trichophagia with no history of treatment. Routine laboratory results are detailed in Table 1. The patient was diagnosed with perforation peritonitis with shock.

**Table 1:** Laboratory investigations Hb: hemoglobin, TLC: total leucocyte count, Na+: sodium

Parameter	Result	Reference Value
Hb	9.8 g/dl	11.6-15.0
TLC	43,000/µL	4000-11,000
Platelet	186,000/µL	150,000-400,000
Blood glucose	87 mg/dl	70-110
Serum Na+	146 mmol/L	145-155
Lactate	6.1 mmol/L	<2.2
Serum creatinine	2.2 mg/dl	0.6-1.2
Serum albumin	3.8 g/dl	3.5-5.5

**Figure 2:**
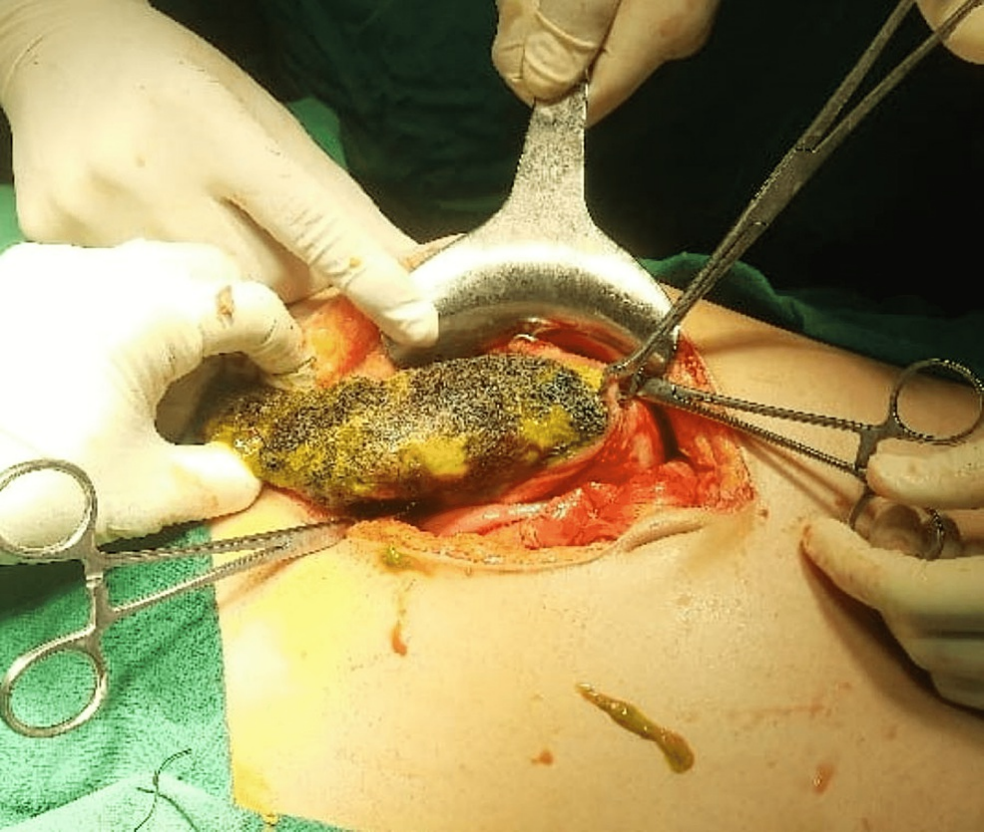
Intra-operative picture showing perforation with trichobezoar in lumen of stomach

**Figure 3:**
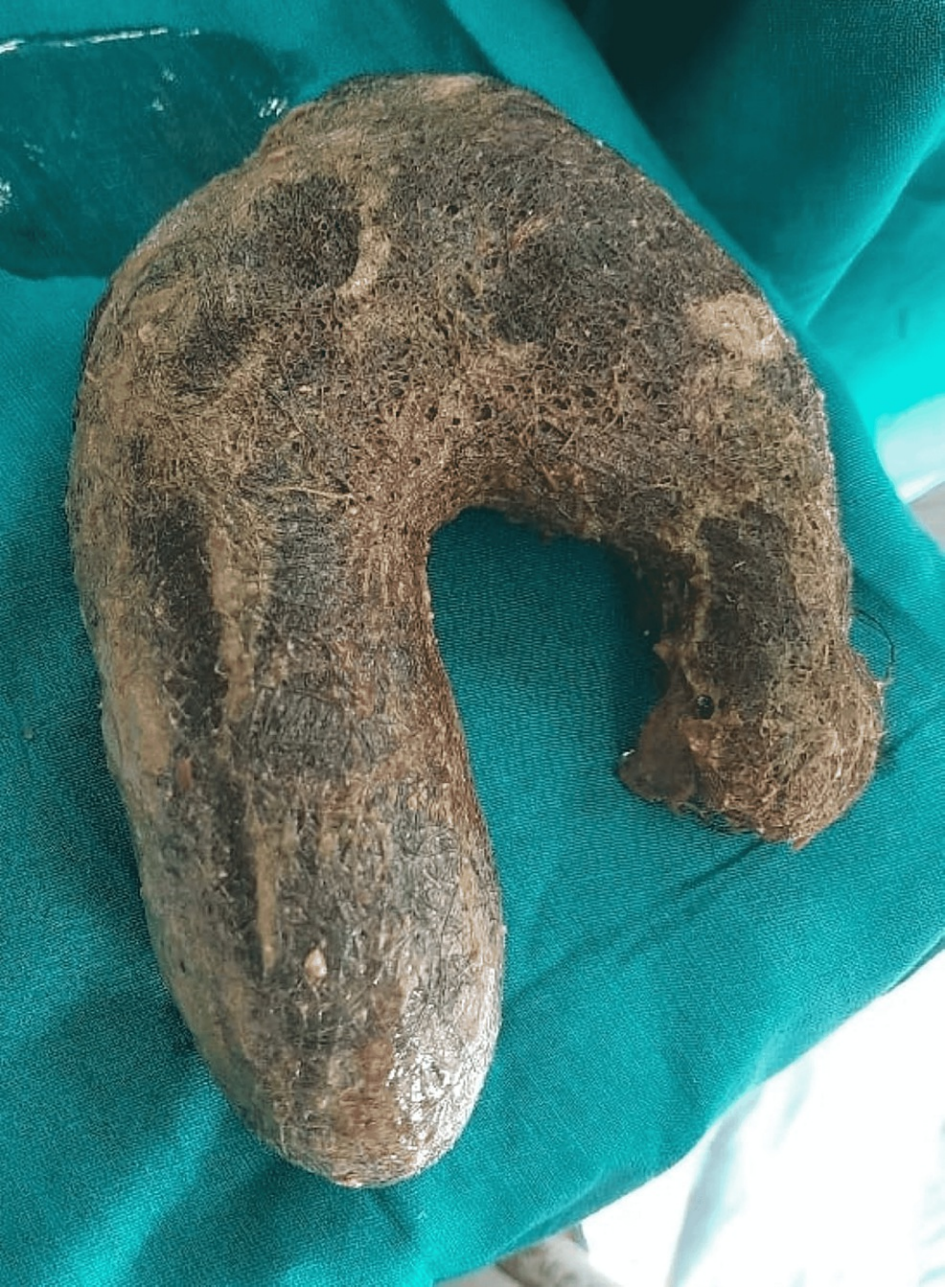
Trichobezoar size 15 cm × 8 cm extracted from lumen of the stomach

Initially, an abdominal drain was placed in the peritoneal cavity under local anaesthesia, which drained the 2 litres of purulent fluid, and when the patient’s parameters improved, she was taken for exploratory laparotomy. Intra-operative findings (Figure 2) revealed a 2 cm × 2 cm perforation over the anterior surface of the stomach body more towards lesser curvature with hair protruding through the perforated part. On exploration, a 15 cm × 8 cm trichobezoar (Figure 3) was retrieved from the lumen of the stomach.

**Figure 4:**
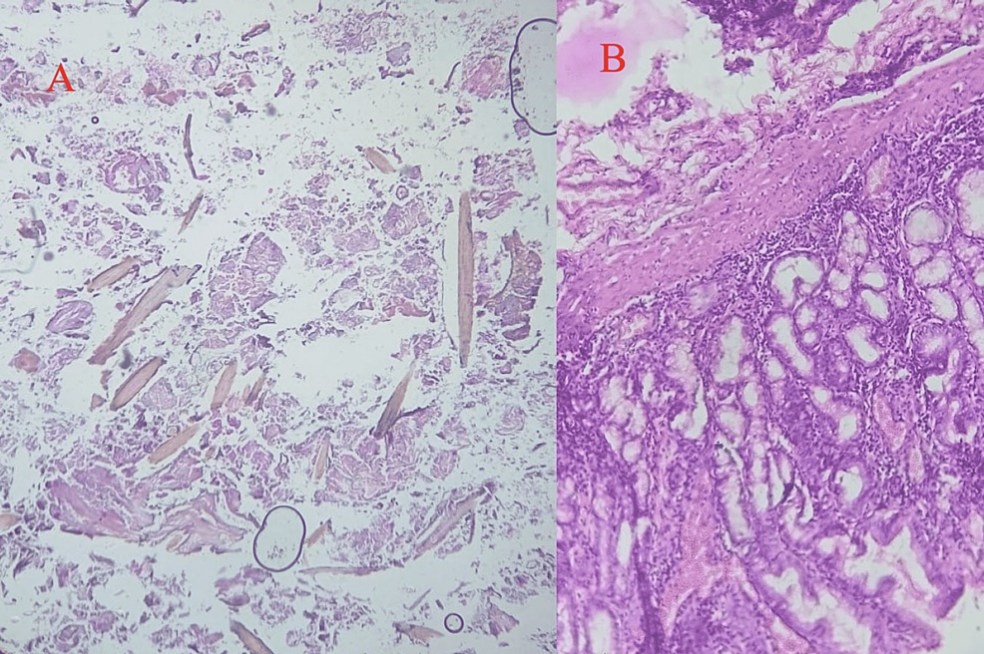
Photomicrographs of gastric biopsy suggestive of inflammation with no specific etiology. Hematoxylin and Eosin stain (×100)

A thorough peritoneal lavage was done with a copious amount of normal saline and the gastric wound was repaired with a double-layer technique, with an inner full thickness and an outer seromuscular layer of 2-0 vicryl and silk suture, respectively. An omental patch was fixed over the gastrostomy site after taking a biopsy from the margins. Abdominal closure was then done. The patient was shifted to the surgical ICU post-operatively. The histopathological report suggested inflammation with no specific aetiology (Figure 4A-4B). During the post-operative period, the patient was managed in the intensive care unit. The patient had CO_2_ retention, serum procalcitonin was >100 ng/ml, and D-dimer was >10,000 ng/ml. She could not be weaned off the ventilator. The tracheostomy was done on post-operative day 7 and the patient succumbed on post-operative day 10 despite all the intense efforts.

## Discussion

A bezoar is a ball of foreign stuff or material in the gastrointestinal tract (GIT) and is called a trichobezoar if composed of hairs. Other bezoars include phytobezoars, pharmacobezoars, or lectobezoars if they are made up of vegetables, medicines, or formulated milk, respectively. Although the usual patient is a teenage girl, trichobezoar has been reported in people of all ages. They are virtually invariably linked to trichotillomania (hair-pulling) and trichophagia (hair swallowing), as well as other psychiatric diseases such as obsessive-compulsive disorder, depression, and body dysmorphic syndrome.

The patient is usually asymptomatic unless the trichobezoar attains a substantial size, which can present as abdominal pain, intestinal obstruction, vomiting, loss of appetite, and weight loss [9]. Our patient presented with abdominal pain and obstipation. The most common complication is perforation of the stomach with an incidence of 10.1%, followed by intussusception, pancreatitis, and cholangitis, seen in 1.85%, 0.92%, and 0.92%, respectively [10]. In a case series, Mirza et al. found that 15 patients (88%) reported abdominal discomfort and vomiting, and 8 (47%) had abdominal distension. One patient had a stomach perforation and rectum bleeding and died in the postoperative period [11]. A 30-year-old woman had a stomach perforation with trichobezoar, which was treated with a laparotomy and led to an uneventful recovery [12].

Trichobezoars are diagnosed with imaging such as USG and CT scans. Ultrasonography is effective and easily available with no radiation risk to the patient, but a CT scan is more descriptive and accurate in describing the bezoar in terms of its size, exact location, extent, and any complications associated with it. Endoscopy is used to make a definitive diagnosis [13]. Our patient was presented with an acute emergency with abdominal distension, obstipation, and shock. In addition, a plain erect radiograph of the abdomen showed free air under the right dome of the diaphragm, indicating some hollow viscus perforation, so in view of the emergency, exploration was done without any advanced imaging, such as a CT abdomen, and was found to have gastric perforation caused by a long-standing trichobezoar.

The exact pathophysiology of perforation in trichobezoar is yet to be established, but the pressure necrosis and gastric mucosal irritation of an enlarging hairball could be the reason. The hairball is obstructing the sufficient entry of food, which is responsible for weight loss, further malnutrition, and hypoalbuminaemia. This hypoalbuminaemia can be a sequela of malnutrition as well as protein-losing enteropathy. Considering this mechanism, the gastric wall becomes less elastic and vulnerable to pressure injuries imparted by enlarging and big trichobezoar [14]. Additionally, hypovolemia adds to less perfusion of the gastric mucosa, so the healing process is delayed for minor mucosal injuries [15]. The lesser curvature of the stomach is more susceptible to ischaemic and pressure injuries as the blood supply to this part is formed by the small and tortuous submucosal arterial plexus, which makes this part more prone to ischaemia/ulceration and so the perforation of the stomach.

Additionally, in the case of Rapunzel syndrome, where peristalsis works against the mechanical obstruction, it results in the weakening of the gastric wall, which could be a reason for gastric perforation. Eventually, the perforation in the trichobezoar is a multifactorial entity, such as gastric wall weakening through mechanical compression and other irritative mechanisms and poor healing ability.

## Conclusions

Acute abdomen in hollow viscus perforation is an emergency where the saving of time is crucial to avoid spillage of visceral contents and to avoid peritonitis, shock, and even mortality. Gastrointestinal perforation and peritonitis in association with trichobezoar are rare entities, so if a patient has some kind of psychiatric disorder with patchy alopecia due to a habit of trichotillomania, they require close watch if they have abdominal pain since it could be due to perforation of the GI tract. Since we lost our patient as a result of the delayed presentation and other complications, the aim of presenting this case is to highlight the early identification of life-threatening complications of trichobezoar, which can save the lives of such psychiatric and ignorant patients if identified beforehand. Therefore, proper clinical history and early surgical intervention play a vital role in reducing morbidity and mortality in these patients.
